# Supplementary material for: Nucleotidyltransferase toxin MenT extends aminoacyl acceptor ends of serine tRNAs to control Mycobacterium tuberculosis growth
Source: Nat Commun. 2024 Nov 6;15:9596. doi: 10.1038/s41467-024-53931-w (PMC11541572; doi:10.1038/s41467-024-53931-w)
Supplement: Supplementary file 1 — Supplementary Information [file 41467_2024_53931_MOESM1_ESM.pdf]

SUPPLEMENTARY INFORMATION FILE

Nucleotidyltransferase toxin MenT extends aminoacyl acceptor ends of serine tRNAs to control *Mycobacterium tuberculosis* growth

Xibing Xu, Roland Barriot, Bertille Voisin, Tom J. Arrowsmith, Ben Usher, Claude Gutierrez, Xue Han, Carine Pagès, Peter Redder, Tim R. Blower, Olivier Neyrolles, Pierre Genevaux

This file contains 6 Supplementary Figures.

a

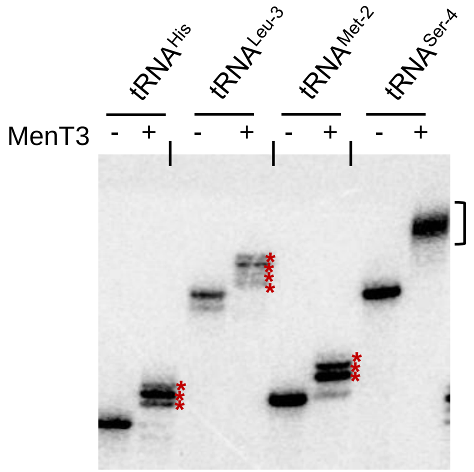

b

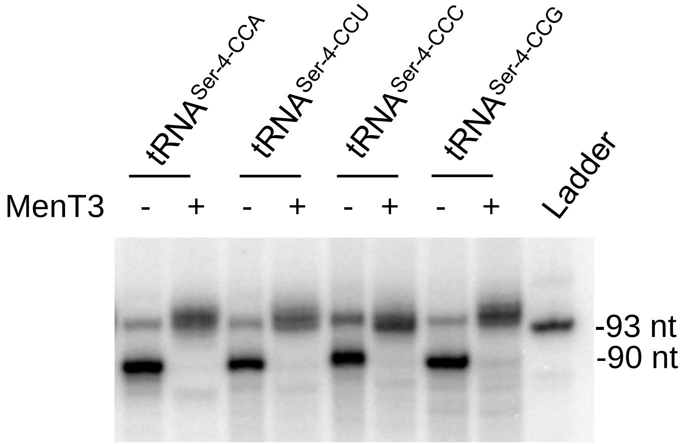

Supplementary Figure S1: Data extended from Figure 2. **a** Purified α-32P labeled tRNA^{Ser-4} and tRNA^{Met-2}, tRNA^{His} and tRNA^{Leu-3} were incubated in the presence of 1 mM CTP with MenT3 (0.2 μM) at 37 °C for 5 min, separated on a 10% urea gel and revealed by autoradiography. **b** Purified α-32P labeled tRNA^{Ser-4} with mutations in the 3′ adenosine of the CCA end were incubated in the presence of 1 mM CTP with MenT3 (0.2 μM) at 37 °C for 5 min, separated on 10% urea gel and revealed by autoradiography. Representative results of triplicate experiments are shown. All the tRNA were prepared by in vitro transcription with homogeneous 3′-ends. Source data are provided as a Source Data file.

a

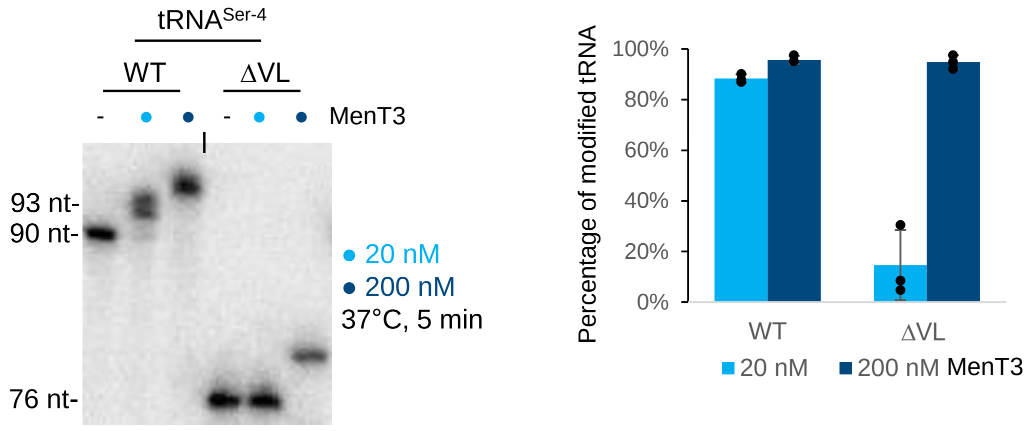

b

Contacts		tRNA^Ser-4 VL (pLDDT)	tRNA^Leu-3 VL (pLDDT)
MenT3	K3	G49 (81.3/91)	C48 (61.1/69.9)<br>U49 (55.6/65.1)
	Y5	C48 (95.9/94.2)<br>G49 (96.1/91.8)	C47 (90.3/74.7)<br>C48 (91/69.1)
	T10	G46 (89.9/92.2)<br>A47 (90.7/93.2)	<50
	N11	G46 (90.9/90.8)<br>A47 (91.8/93.9)	<50
	R13	G46 (73/92.6)<br>A47 (86.8/93.8)	<50
	S14	A47 (97.5/93.7)<br>C48 (97/94.3)	C47 (89.9/74.8)
	D17	A47 (94.3/93.8)<br>C48 (96.7/94)	<50
	R18	A47 (97.2/93.8)<br>C48 (97.794.3)	C47 (93.1/74.6)
	Q21	C48 (90.3/93.3)<br>G49 (88.5/92)	C47 (73.1/74.9)

c

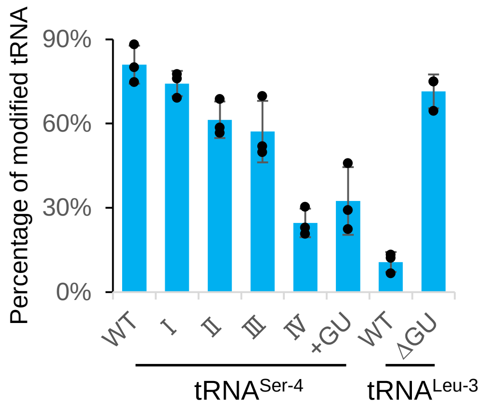

d

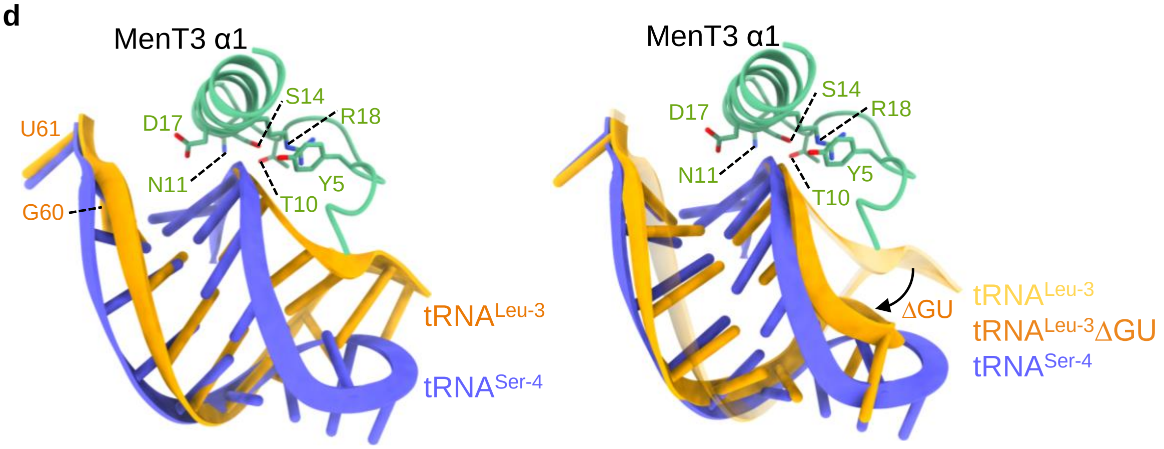

Supplementary Figure S2: Impact of the variable loop of $tRNA^{Ser-4}$ on the modification by MenT3. **a** Purified [α-32P]-labeled $tRNA^{Ser-4}$ or $tRNA^{Ser-4}$ ΔVL were incubated with 20 or 200 nM MenT3 in the presence of 1 mM CTP at 37 °C for 5 min. The tRNAs were separated on a 10% urea gel and visualized by autoradiography, as shown on the left panel. The percentage of modified tRNA is represented on the right panel as the mean ratio of modified products to total tRNA, derived from three independent experiments. Errors and P-values were obtained by STDEV and T.test, respectively. P-values of $tRNA^{Ser-4}$ WT compared to ΔVL are 0.00079 and 0.65491, when 20 nM and 200 nM MenT3 are used in the reaction, respectively. **b** Proposed interaction surface between MenT3 and the VL of $tRNA^{Ser-4}$ or $tRNA^{Leu-3}$ using Alphafold3. The Alphafold3 model results were submitted to PREDICTOMES-AlphaFold 3 Analysis Tool (BETA), the pLDDT scores from MenT3-$tRNA^{Ser-4}$ or $tRNA^{Leu-3}$ VL are presented in the table. **c** The percentage of modified tRNA is represented as the mean ratio of modified products to total tRNA, derived from three independent experiments from Fig. 3c. P-values of $tRNA^{Ser-4}$ WT compared to mutant I, II, III, IV, +GU, of $tRNA^{Leu-3}$ compared ΔGU are 0.22267, 0.02199, 0.03266, 0.00032, 0.00368 and 0.00013, respectively. **d** Model of the MenT3 and tRNA complex generated using AlphaFold3. The structure illustrates the binding interaction between MenT3 and $tRNA^{Ser-4}$, $tRNA^{Leu-3}$ or $tRNA^{Leu-3}$ ΔGU as processed in Fig. 3a. Source data are provided as a Source Data file.

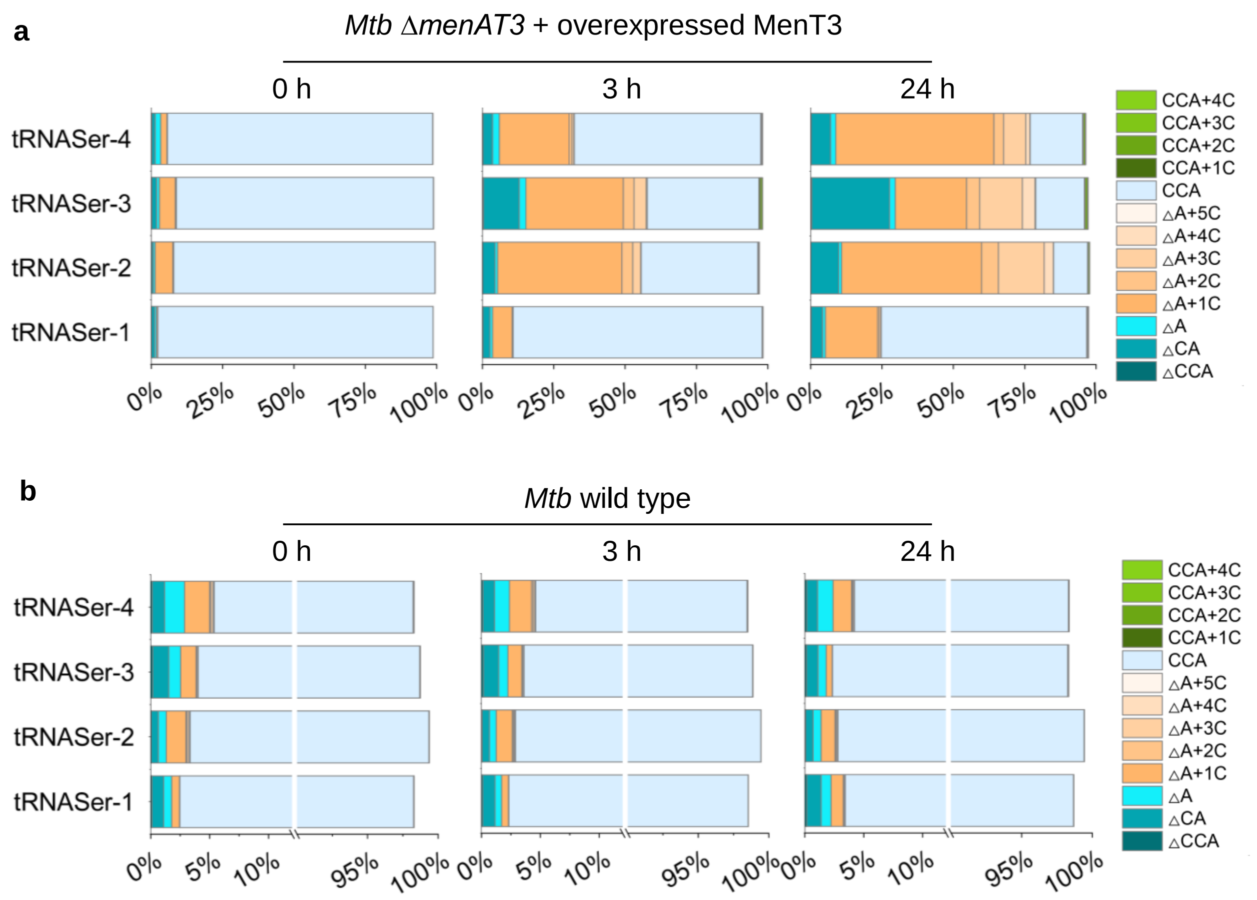

Supplementary Figure S3: A detailed analysis of the tRNA seq data of the 3′-end of tRNA$^{Ser\text{-}1,\ 2,\ 3\ and\ 4}$ of *M. tuberculosis* wild-type H37Rv strain and its isogenic mutant Δ*menAT3* expressing MenT3. The data were mean analyzed from three independent experiments. Source data are provided as a Source Data file.

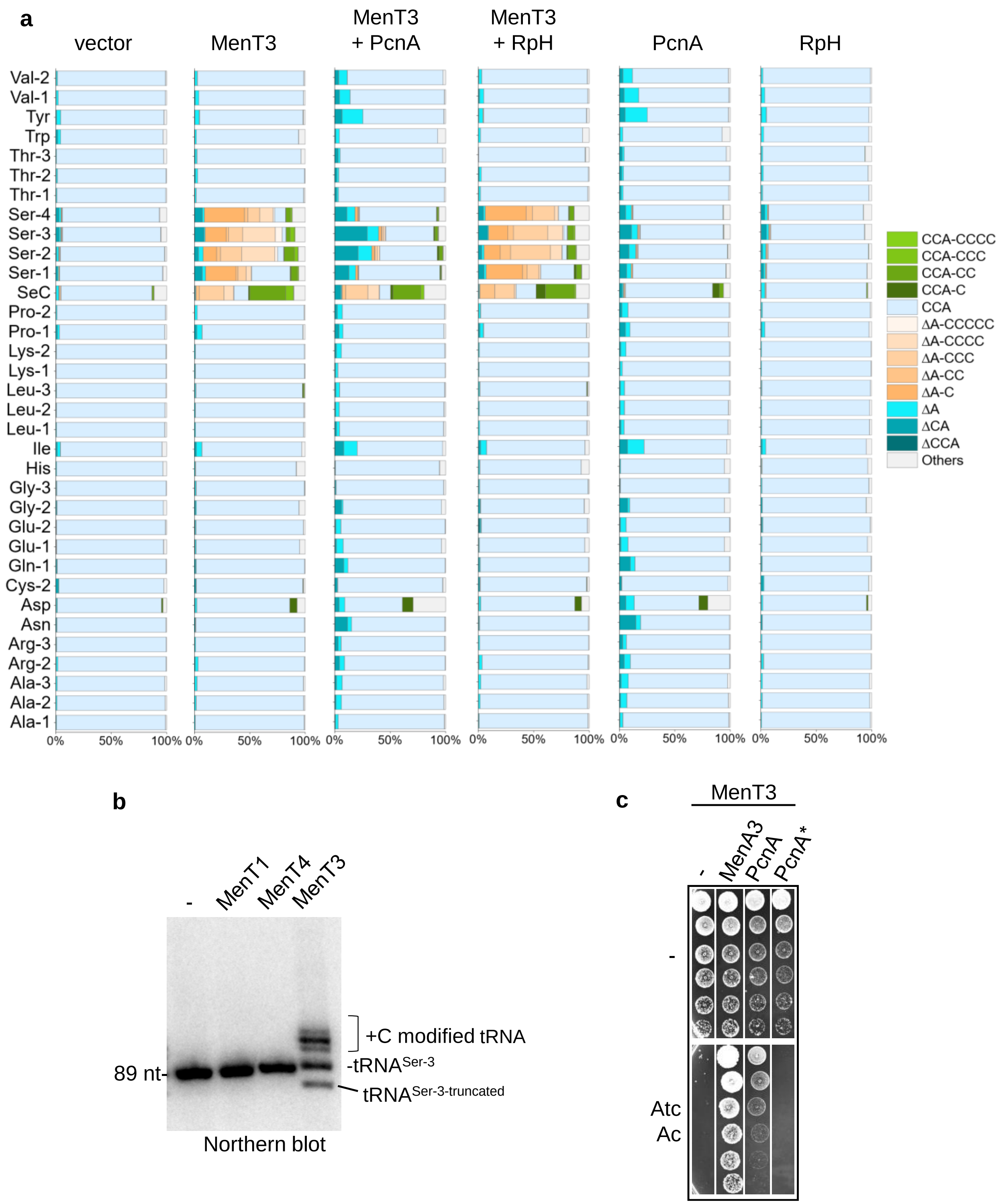

Supplementary Figure S4: a tRNA-seq analysis in *M. smegmatis* overexpressing MenT3, MenT3+PcnA, MenT3+ RpH, PcnA or RpH. tRNA-seq analysis of RNA extracts from *M.*

smegmatis co-transformed with various plasmid combinations: pGMC/pLAM, pGMC-MenT3/pLAM, pGMC-MenT3/pLAM-PcnA, pGMC-MenT3/pLAM-RpH, pGMC/pLAM-RpH, and pGMC/pLAM-PcnA. Cultures of transformants were grew until an OD600 of 0.1 in fresh LB medium and induced with 100 ng ml^{-1}Atc and 0.2% Ace for 3 hours at 37°C. RNA was extracted and analyzed by tRNA-seq. Shown is a mean representation from triplicate experiments. *M. smegmatis* containing empty vector or pGMC-menT3 co-transformed with pLAM-vector, -PcnA, or RpH were individually grown at 37 °C in LB supplemented with 0.05% Tween 80. When the OD_{600} reached to 0.1, the anhydrotetracycline inducer (Atc, 100 ng ml^{-1}) was added and cells were collected after 3 h incubation at 37 °C. Total RNA was extracted and tRNA-seq was performed as in Fig. 3. Results are from two independent experiments (Duplicate is shown in datasheet file). **b** The *M. smegmatis* RNA extracts from panel a were subjected to northern blot analysis using a probe against $tRNA^{Ser\text{-}3}$ (labelled by γ-32P) and revealed by autoradiography. The results revealed the presence of elongated $tRNA^{Ser\text{-}3}$ as well as a truncated $tRNA^{Ser}$ was observed on a TBE-Urea gel, likely corresponding to the $tRNA^{Ser}$-CΔCA identified in the tRNA-seq data. Representative results of duplicate experiments are shown. **c** Related to Fig. 5a, PcnA putative NTase domain mutant DLD (59-61)-AAA (PcnA*) abolishes its anti-MenT3 activity. Shown are representative results from triplicate experiments. Source data are provided as a Source Data file.

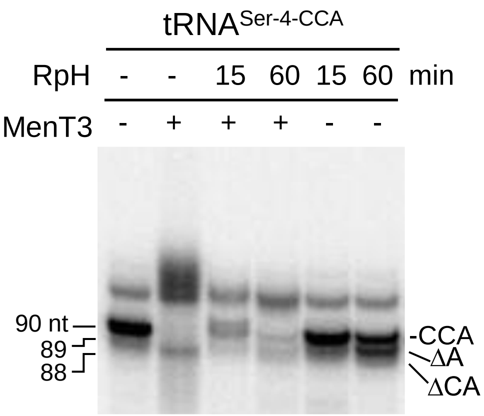

Supplementary Figure S5: RpH-mediated response to MenT3 modifications in vitro. tRNA repair assay used to test RpH of *M. tuberculosis*. α-32P labelled tRNA^{Ser-4} was incubated with MenT3 (0.2 µM) or without for 5 min at 37 °C and the modified tRNA was subjected to repair by RpH (10 µM) for 15 or 60 min at 37 °C, the samples were separated on a 10% urea gel and revealed by autoradiography. Representative results of triplicate experiments are shown. Source data are provided as a Source Data file.

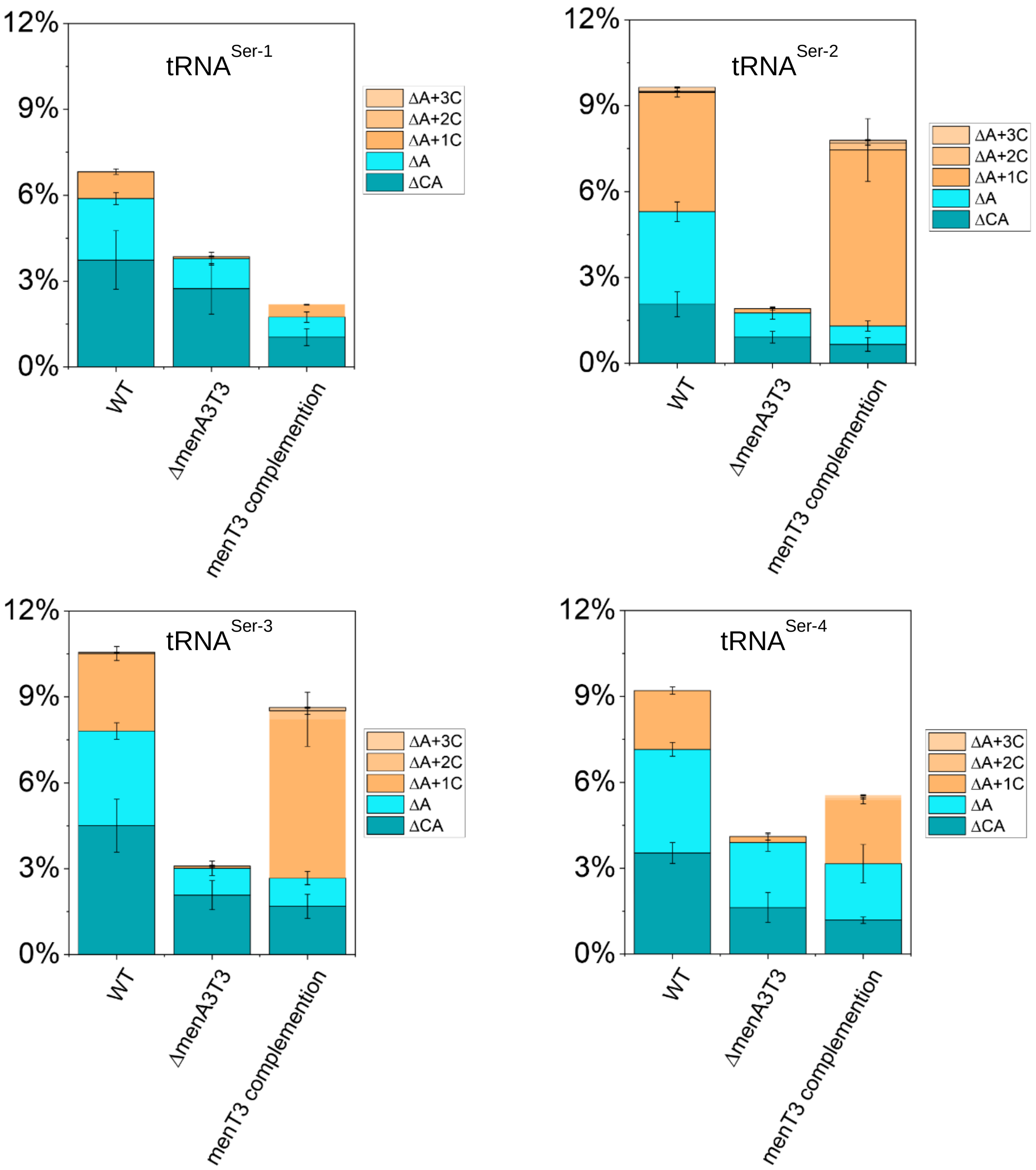

Supplementary Figure S6: Details of the modifications found for four tRNA^{Ser} isoacceptors were analyzed among wild-type *M. tuberculosis*, Δ*MenAT3* and menT3 complementation strain. The data shown as means of three independent experiments. Source data are provided as a Source Data file.
