# Supplementary material for: Nucleotidyltransferase toxin MenT extends aminoacyl acceptor ends of serine tRNAs to control Mycobacterium tuberculosis growth
Source: Nat Commun. 2024 Nov 6;15:9596. doi: 10.1038/s41467-024-53931-w (PMC11541572; doi:10.1038/s41467-024-53931-w)
Supplement: Supplementary file 3 — Description of Additional Supplementary Files [file 41467_2024_53931_MOESM3_ESM.pdf]

Description of Additional Supplementary Files

Supplementary Data 1: List of primers and sequences used in this work
